# American Society of Anaesthesiologists physical status classification

**DOI:** 10.4103/0019-5049.79879

**Published:** 2011

**Authors:** Mohamed Daabiss

**Affiliations:** Department of Anaesthesia, Riyadh Armed Forces Hospital, Riyadh, Kingdom of Saudi Arabia

**Keywords:** Anaesthesia, ASA, physical status classification, preoperative assessment

## Abstract

Although the American Society of Anaesthesiologists’ (ASA) classification of Physical Health is a widely used grading system for preoperative health of the surgical patients, multiple variations were observed between individual anaesthetist’s assessments when describing common clinical problems. This article reviews the current knowledge and evaluation regarding ASA Classification of Physical Health as well as trials for possible modification.

## INTRODUCTION

In 1941, The American Society of Anaesthesiologists (ASA) asked a committee of three physicians: Meyer Saklad, Emery Rovenstine and Ivan Taylor to study, examine, experiment and devise a system for the collection and tabulation of statistical data in anaesthesia to allow anaesthesiologists to record the overall health status of a patient prior to surgery and, thereby, allow patients outcome to be stratified by a general assessment of illness severity.[1] While their mission was to determine predictors for operative risk, they quickly dismissed this task as being impossible to devise. ASA proposed the physical status classification of preoperative patients for anaesthetic risk assessment in 1963.[2]

The ASA score is a subjective assessment of a patient’s overall health that is based on five classes (I to V).

- Patient is a completely healthy fit patient.
- Patient has mild systemic disease.
- Patient has severe systemic disease that is not incapacitating.
- Patient has incapacitating disease that is a constant threat to life.
- A moribund patient who is not expected to live 24 hour with or without surgery.

E. Emergency surgery, E is placed after the Roman numeral.

Since inception it has been revised on several occasions and an ‘E’ suffix was included denoting an emergency case. Being simple and widely understood, ASA score also has been used in policy making, performance evaluation as an easy tool for audit, resource allocation, reimbursement of anaesthesia services and frequently is cited in clinical research as well.

## CORRELATION WITH OUTCOME

Associations between ASA scores and specific surgical complications and outcomes have been reported in the literature. It was considered to be an important tool predicting short- and long-term outcome in patients undergoing hepatic resections and as a useful tool in adapting individual therapeutic strategies in order to improve surgical outcome in patients with primary and secondary hepatic malignancies.[3]

The rate of postoperative complications was found to be closely related to the ASA class (ASA score I = 0.41/1,000; scores IV and V = 9.6/1,000) and with emergency surgeries (ASA I = 1/1,000 increases to 26.5/1,000 in classes IV and V).[4]

The specific correlation of ASA scores with operating times, hospital length of stay, postoperative infection rates, overall morbidity and mortality rates following gastrointestinal, cardiac, and genitourinary surgery has also been extensively studied.[5–9] Moreover, the predictive impact of the ASA classification was studied in a prospective study with 295 consecutive total abdominal hysterectomy patients and it was reported that ASA scores are correlated with total blood loss during surgery.[10] In particular, ASA score III is a predictor of greater blood loss, and therefore transfusion units required as compared to lower ASA class patients. Another prospective study of 168 patients admitted to geriatric hip fracture service found that an ASA score of III or more is a predictive factor of postoperative delirium.[11]

In addition, the ASA score had been found in some studies to be a strong predictor of postoperative resource utilization and mortality in numerous surgical fields. It was significantly related to the incidence of postoperative death in a group of 3,438 elective total hip and total knee arthroplasty (TKA) patients with class III patients were more likely to encounter postoperative death as compared to patients with lower ASA scores.[12]

Finally, Wolters and his colleagues examined the strength of association between ASA physical status classification and perioperative risk factors and postoperative outcome in a prospective study of 6301 surgical patients in a university hospital using univariate analysis and calculation of the odds ratio of the risk of developing a postoperative complication by means of a logistic regression model.[13] Univariate analysis showed a significant correlation (*P* < 0.05) between ASA class and perioperative variables (intraoperative blood loss, duration of postoperative ventilation and duration of intensive care stay), postoperative complications and mortality rate. Univariate analysis of individual preoperative risk factors demonstrated their importance in the development of postoperative complications in the related organ systems. Estimating the increased risk odds ratio for single variable, we found that the risk of complication was influenced mainly by ASA class IV (risk odds ratio = 4.2) and ASA class III (risk odds ratio = 2.2), and they conclude that ASA physical status classification was a predictor of postoperative outcome.

## DISAGREEMENTS AND INCONSISTENCY WITH RATING

Nevertheless, considerable variation in the ASA classification allocation has been reported in previous studies as it neither does consider the patient age, sex, weight, and pregnancy nor the nature of the planned surgery, the skill of the anaesthetist or surgeon, the degree of pre-surgical preparation or the facilities for postoperative care.[14–16] The definitions are based on severity of disease and may result in inconsistent application. The measure of surgical complexity in the ASA classification system is less clear. The terms minor, intermediate and major are used to categorize the complexity of surgery. However, the assumption is that these definitions are intuitive and self-explanatory.

The word ‘systemic’ in ASA classification creates a lot of confusion. For example, heart attack (myocardial infarction), though grave, is a ‘local’ disease and is not a ‘systemic’ disease, so a patient with recent (or old) heart attack, in the absence of any other systemic disease, does not truly fit in any category of the ASA classification, yet has poor post-surgery survival rates. Similarly, cirrhosis of the liver, COPD, severe asthma, peri-nephric abscess, badly infected wounds, intestinal perforation, skull fracture, etc are not systemic diseases. These, and other severe heart, liver, lung, intestinal or kidney diseases, although they greatly affect physical status of patient and risk for poor outcomes, cannot be labelled as ‘systemic disease’ (which means a generalized disorder of the whole body like hypertension or diabetes mellitus). Local diseases can also change physical status but have not been mentioned in ASA classification.

A secondary issue is that most facilities do not provide a full range of services in their operating suite services and therefore divide their caseload into major and minor cases. This division may not reflect the commonly held assumptions about major and minor, but reflect a split of local caseloads.[15]

The ASA Physical Status Classification had been previously tested for consistency of use by anaesthetists. While, the length of hospital resource utilization was not predicted by the preoperative ASA score of elective TKA patients, but also similar anaesthesia costs, operating room costs, total hospital costs, and length of stay (LOS) was found in 100 TKA patients of ASA scores I to III.[17] Others have found ASA score to correlate with LOS following other types of surgery.[9,18]

The preoperative ASA score was not found to have a predictive quality towards morbidity and mortality after major abdominal surgery.[19] Dr. Owens clarified why the ASA classification system does not predict risk, saying, ‘The kind of operative procedure is not a part of the classification system because a physical status, patient is still in that status if scheduled for an excision of a skin lesion with monitored anaesthesia care or if scheduled for a pancreatectomy with general anaesthesia. The operative risk is different because of the surgery, but the physical condition of the patient is the same preoperatively’.[20]

Different authors give different versions of this ASA definition. It is because this classification is vague and far from perfect. Many authors try to explain it on the basis of ‘functional limitation’ or ‘anxiety’ of patient which are not mentioned in the actual definition. However, inconsistency of grading between anaesthetists has been demonstrated in studies using hypothetical adult patient scenarios. One study reported several sources of variability between anaesthesia providers including smoking, pregnancy, nature of the surgery, potential difficult airway, and acute injury.[21] Another study using a questionnaire depicting 10 hypothetical patient cases was sent to 249 randomly selected specialists and non-specialists anaesthesiologists working in university teaching and non-teaching hospitals in Finland.[22] They found a marked variation in the classification of all the 10 cases: 1 case was classified to all five possible grades (ASA grades I-V). In two cases, there was a significant variation between anaesthesiologists working in university teaching and non-teaching hospitals, while there was no difference in the grading between specialist and non-specialist anaesthesiologists.

In a similar study, age, obesity, previous myocardial infarction, and anaemia provoked controversy. Academic anaesthesiologists rated a greater number identical than did those in private practice.[23] Moreover, when the interrater reliability of the ASA grading system in paediatric anaesthesia practice was investigated, many limitations of the ASA system in paediatric practice were found. Case scenarios involving trauma or airway compromise were associated with greater inconsistency.[24]

However, the published absolute mortality rates of the individual classes showed considerable variation, with 0-0.3% for ASA I, 0.3-1.4% for ASA II, 1.8-4.5% for ASA III, 7.8-25.9% for ASA IV and 9.4-57.8% ASA V.[25] This variation may be explained by differences in assessment of the patient’s ASA physical status, patient population, sample size, operations performed and duration of postoperative monitoring. The latter is particularly important, as some of the older studies included only deaths occurring within the first 48 h or within the first 7 days postoperative, while none covered the hospital stay. Thus these studies missed almost 50% of postoperative deaths occurring after the 7^th^ postoperative day. Often these limits are placed to assess the possible role of anaesthesia in postoperative mortality.

## TRIALS OF RATING MODIFICATION

Thus, since the introduction of the ASA score, several studies have highlighted disagreements and inconsistency of ratings, while others tried to find a modification to improve rating consistency. Atilio and colleagues had suggested the addition of a modifier for pregnancy to the current classification.[14] As the pregnant patient presents physiologic disturbances that may increase her anaesthetic risk and require special attention in her anaesthetic management; these factors are not included in a disease state stratification.[26] They evaluated the use of the G modifier similar to the modifier, E; for emergency cases and found that a number of anaesthesiologists reduced the rating when given the option of the G modifier.

Moreover, the modifier allows the rater to concentrate simply on the parturient’s concomitant diseases, as well as to communicate the preoperative status of a patient with precision and to allow a more precise classification of patient groups, more effective communication between professionals and more accurate stratification of patient groups for statistical or outcome analysis.[14]

Tomoaki and Yoshihisa reported that it is difficult to estimate whether the class II patients have an accurate risk ranging from mild to moderate-severe systemic disorders since the ASA class II is very broad and does not accurately reflect the patients’ risk.[15]

They assessed 1933 patients scheduled for surgical procedures both by 5-grade ASA physical status protocol and by their new 7-grade preoperative status assessment dividing classes I and II into *a* and *b*.

**Table:** 

Class I:	I*a* :	Normal healthy patient.
	I*b* :	Patient with mild systemic disease.
		Normal healthy patient with anaesthetic or operative risk.
Class II:	II*a* :	Patient with moderate systemic disease. Patient with mild systemic disease with anaesthetic or operative risk.
	II*b* :	Patient with moderate to severe systemic disease that does not limit activity. Patient with moderate systemic disease with anaesthetic or operative risk.

## TYPICAL OPERATIVE AND ANAESTHETIC RISK FACTORS EXCLUDING PHYSICAL STATUS FOR REVISED ASSESSMENT

### Operative factors

Cardiovascular operations, thoracotomy/sternotomy, thoracoscopic operations, operation in airway. Expectation of severe bleeding, prolonged operation, brainstem operation, prolonged postoperative controlled ventilation, pregnancy except caesarean section, etc.

### Anaesthetic factors

Special position, expectation of difficult intubation or difficult intravenous cannulation, susceptibility of malignant hyperthermia, full stomach, one lung ventilation, refusal of blood transfusion, not in operating room, etc.

Half point was added when each of the specific risk factors in anaesthetic and surgical categories was present. In this new 7-grade classification, they classified that the grade 1 was to grade I*a* (no risk of life), the grade 1.5 was to grade I*b* (almost no risk of life), the grade 2.0 was to II*a* (light risk of life) and the grade 2.5 was to II*b* (middle risk of life). There were no changes in grades 3 (heavy risk of life), 4 (very dangerous risk of life) and 5 (almost death risk of life). Postoperative complications within 1 week in operated patients were collected from their medical records.

The number of patients in the revised classification gradually decreased from grades 1a to 3. In contrast, the number of patients in the ASA classification was not evenly distributed in grades 1 to 3. The incidence of intra- and postoperative complications in both the ASA and revised classifications gradually increased from grades 1 to 3 and 1a to 3, respectively. However, the largest numbers of patients in the ASA and revised classifications were distributed in grade 2 and grades 1b and 2a, respectively. In terms of emergency cases, the largest numbers of patients in the revised classification were distributed in grades 1b and 2a, while those in ASA classification were mostly in grade 2. The distribution of complication incidence in both the ASA and the revised classification showed a gradual increase from grades 1 to 5, whereas the largest numbers of patients in the ASA classification were distributed in grades 2 and 3, and the largest numbers of patients in the revised classification were distributed in grades 2a, 2b and 3. The authors reported that this revised classification is practical and reasonable, because the prediction of intra- and postoperative complications with this assessment was more accurate than that with the conventional ASA classification. Besides, this classification could be acceptable for most practitioners, because it is principally based on the ASA physical status.[15]

## SUMMARY

This review has presented diverse opinions regarding ASA Classification of Physical Health. Although ASA scoring stands to assess the global anaesthetic conditions for patients, it does not exactly assess the periopertive conditions for recent practical use. ASA physical status (7-grade) can provide a better grading outcome for predicting the incidence of intra- and postoperative complications in surgical patients. The usefulness of the new 7-grade classification including anaesthetic and/or surgical risk categories in routine anaesthesia practice should be evaluated by multicenter study with the conventional ASA.
